# Attention Matters: Pitch vs. Pattern Processing in Adolescence

**DOI:** 10.3389/fpsyg.2013.00333

**Published:** 2013-06-10

**Authors:** Elyse S. Sussman

**Affiliations:** ^1^Departments of Neuroscience and Otorhinolaryngology-HNS, Albert Einstein College of Medicine, Bronx, NY, USA

**Keywords:** attention, auditory, adolescents, mismatch negativity, pitch, pattern processing

## Abstract

From the moment we wake up, we are flooded with more sensory inputs than we can possibly process. Selective attention mechanisms serve to limit the sensory onslaught, while facilitating the ability to perform everyday tasks. However, not much is known about the typical development of selective attention mechanisms during childhood even though impairments of attention are commonly noted in neurodevelopmental disorders. The current study focuses on a transitional time in child development, adolescence, to determine in what way specific auditory tasks have a modulatory effect on underlying brain activity to facilitate behavioral goals. Neural mechanisms of selective attention were tested through auditory pitch and pattern perception, using a measure of event-related brain potentials (ERPs) called the mismatch negativity (MMN). Sounds with a regular five-tone pattern were presented in three conditions. The conditions differed only in how participants were instructed to listen to the sounds. Focus was either on the pitch of the sounds, the pattern of the sounds, or on a close-captioned movie. Even though the sound input was identical in all conditions, task-specific modifications were manifest in the MMN evoked by the deviant sounds embedded in the test sequences. The results demonstrate that in adolescence, as in adults, selective attention alters neural activity specific to performance goals, thus indicating specific neural adaptation modulated by behavior.

## Introduction

“My experience is what I agree to attend to.” (James, 1890, p. 402).

More sensory information reaches us that we can possibly deal with. One of the goals of attention is to limit the amount of information we perceive. When we choose to attend to certain parts of the sensory input it changes our experience of the environment. If I now ask you to direct your attention to your shoes, you can feel the pressure of them against your feet. Though, you probably didn’t notice your shoes before I asked you to attend to them even though the sensory information was always available to you. Active selection initiates an interaction between the input (bottom-up processes) and task goals (top-down influences) (Treue, 2001; Beck and Kastner, 2009; Shapiro and Miller, 2011; Miller and Buschman, 2012), modulating the flow of information. This effectively alters our experience of the environment by putting into focus what we choose (or select) to attend to. Attention is adaptive.

It is well known that selecting among inputs can bias underlying neural activity associated with it (Hubel et al., 1959; Desimone and Duncan, 1995; Shomstein and Yantis, 2004). In the biased-competition model of visual selective attention (Desimone and Duncan, 1995), for example, when there are multiple objects in a visual scene, the attended object “wins” the competition for neural representation in favor of the unattended objects. Attention mediates the neural responses, enhancing the activity of the selected object. The indication of such results is that attention acts to facilitate behavioral goals.

The current study took a slightly different focus on selective listening, in which selection was initiated by altering task performance with the same set of sounds. There was one stream of sounds that was fully attended to perform different tasks. The “object” was determined by the task requirements that put different attributes of the same sequence into focus, not by competing bottom-up inputs that could be ignored. Thus, competition was derived fully by the task demand and not by ignoring different parts of the input.

Adolescence is a time of considerable growth in both cognitive and brain functions (Crone, 2009). The ability to selectively attend to the environment has been shown to improve during development (Stuss, 1992). Changes in executive function during this time indicate an increased ability to switch between tasks (Crone et al., 2006), to monitor actions (Ladouceur et al., 2010), and to hold items in memory (Crone et al., 2006; Bunge and Wright, 2007). Concurrently, the brain is undergoing continued developmental changes (Gogtay et al., 2004; Blakemore and Choudhury, 2006; Casey et al., 2008), such as a decrease in gray matter consequential to an increase in synaptic pruning (Sowell et al., 2001; Gogtay et al., 2004). Consistent with continued brain maturation through adolescence, the cortically generated obligatory event-related brain potentials (ERPs) that are measured at the scalp have not yet reached the maturity level of adulthood (Ponton et al., 2000; Gilley et al., 2005; Wunderlich and Cone-Wesson, 2006; Sussman et al., 2008). Thus, this developmental time period is a unique age group for investigation, with little known about the link between brain responses and higher level cognitive skills.

The goal of the current study, in adolescents, was to test task-specific modulation of neural activity resulting from selectively attending to either the pitch or the pattern of a single stream of sounds to determine how selective attention modulates neural activity associated with task goals. That is, the listener is not selecting a subset of the whole information, but rather refocusing attention to different aspects of the sounds by combining or separating elements of a single sound stream to perform a task.

**Figure 1:**
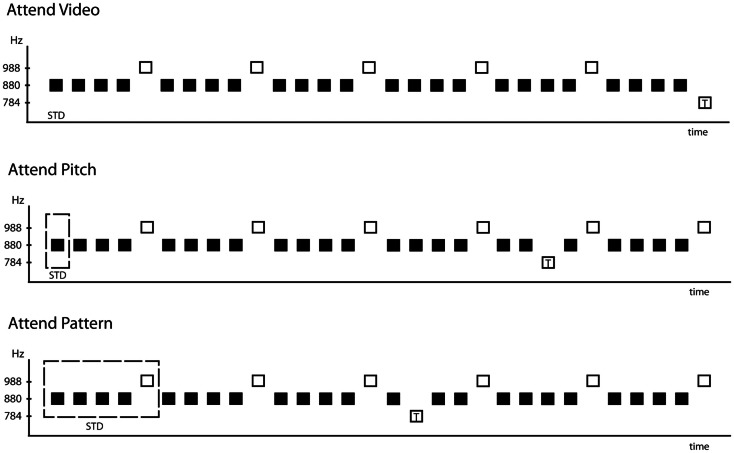
**Schematic of the stimulus paradigm**. The frequency (in Hz) of the tones is indicated on the ordinate and time is represented on the abscissa. The 880 Hz tones, represented by filled black squares, occurred 80%, and the 988 Hz tones, represented by the open squares, occurred 18%. The sound sequence containing the five-tone repeating pattern was presented in all three conditions: Attend-Video (top row), Attend-Pitch (middle row), and Attend-Pattern (bottom row). Target tones are represented by the open square with a “T” inside. The position of the target shown in each condition denotes that the target occurred randomly (2%), whereas the other tones were presented in a fixed order. The dashed square outlines the smallest tonal element needed to perform the task, thus denoting the standard used in the MMN deviance detection process.

To test this, we presented a patterned sequence of sounds and instructed participants to focus on the pitches of the sounds (Attend-Pitch condition), the pattern of the sounds (Attend-Pattern condition), or away from the sounds (Attend-Video condition). The tone pattern in the sequence was determined by two tones occurring in a regularly repeating five-tone pattern (AAAABAAAAB …, where “A” represents a tone of one frequency and B represents a tone of a slightly higher frequency, Figure 1). A third tone (slightly lower in frequency) was presented randomly, and rarely, in the sequence. This tone served as the target so that participants would never be pressing the response key to the B tones that provided the dependent measure. Listening to the pitches required focus on the individual sounds to compare among them, whereas listening to the pattern of sounds involved temporally connecting them to perceive the repeating sequence.

We measured components of the ERPs associated with deviance and target detection. In particular, the mismatch negativity (MMN) component was used to index deviance detection. MMN is particularly useful for addressing the question because (1) MMN provides an index of deviance detection regardless of the direction of attention (Näätänen, 1992; Sussman et al., 2003b; Winkler et al., 2005). Therefore, it could be elicited in all conditions of attention; and (2) MMN is highly context dependent (Sussman and Steinschneider, 2006; Rahne et al., 2007; Sussman, 2007; Rahne and Sussman, 2009), and thus can index how sounds are organized in memory. MMN elicitation is based on tones that are detected as deviant in comparison with tones detected as standard (Näätänen, 1992; Näätänen et al., 2001; Sussman, 2007). Based on our previous studies in adults, we expected that selectively listening to the pitches of the sounds vs. the patterns of the sounds would modulate the neurophysiological response to the B tones in accordance with the task being performed (Sussman et al., 1998, 2002; Sussman and Gumenyuk, 2005).

We predicted that if the B tone was always detected as an infrequently occurring tone of a different frequency than the A tone (focus on pitch), then MMN would be elicited by the B tone in all conditions. In contrast, if the five-tone repeating pattern of the sequence was detected in all conditions (focus on pattern), then no MMN would be elicited by the B tone because it would be held in memory as part of the standard repeating pattern, and not as a deviant. Thus, task modulation of the neural activity to support performance goals would be observed by MMN evoked by B tones only when the pitches of the tones were used to perform the task, but not when the pattern was relevant for the task.

## Materials and Methods

### Participants

Ten adolescents (six males), ranging in age from 13 to 17 years (*M* = 15, SD = 1) participated in the study. All procedures were approved by the Internal Review Board of the Albert Einstein College of Medicine. Parents were reimbursed for their travel expenses and adolescents were given a gift certificate for their participation. After the experimental protocol was explained to them, parents gave informed consent and children gave written assent. All of the children were in their age-appropriate grade in school, passed a hearing screening test (20 dB HL or better from 500 to 4000 Hz), and had no reported history of neurological disorders.

### Stimuli

Three pure tone stimuli, 50 ms duration (5 ms rise/fall time, calibrated to 79 dB peak-to-peak equivalent using a Brüel and Kjaer 2209 sound level meter), were presented with a 575 ms onset-to-onset pace binaurally through insert earphones. The three stimuli differed only in tone frequency (784, 880, and 988 Hz). Two of the tones were presented in a continuously repeating five-tone pattern (AAAABAAAAB …, where “A” denotes the 880 Hz tone and “B” denotes 988 Hz). Thus, the B tone was presented as every fifth tone in the sequence (*p* = 0.20). The 784 Hz tone randomly replaced the A tones (*p* = 0.02), and was the target tone when a task was performed with the sounds (Figure 1).

### Procedures

The patterned tone sequences were presented in three conditions of attention: Attend-Pitch, Attend-Pattern, and Attend-Video. In the Attend-Pitch condition, participants were instructed to listen to the three different pitches of the tones and press the response key when they heard the rarely occurring, lowest-pitched tone. In the Attend-Pattern condition, participants were instructed that there was a five-tone repeating pattern of tones in the sequence and to press the response key when they detected a different pattern that occurred rarely. Consequently, in both task conditions, the target tone was the same rarely occurring lower-pitched tone and only the instruction of when to press the response key differed (i.e., for a pitch change or a pattern change). In the Attend-Video condition, participants were told that they would hear sounds in their ears, to ignore the sounds and watch the captioned video of their choosing. Thus, the patterned sequences were played in all three conditions and only the instructions of how to attend to the stimuli differed (attend to the pitch, to the pattern, or ignore the sounds).

Participants sat in a comfortable chair in an electrically shielded and sound-attenuated booth (IAC, Bronx, NY, USA). Five blocks of 300 stimuli were presented for each of the attend conditions (∼3 min per block) and three blocks of 500 stimuli (∼5 min per block) for the ignore condition. Attend condition blocks were shorter than the Ignore condition blocks to reduce sustained attention effects or fatigue that may occur during longer focused task demands. However, the overall presentation amount was the same in all conditions. About 1155 A tones, 300 B tones, and 45 target tones were obtained in each condition. Total session time was 1.5–2 h, which included time for electrode placement and breaks. Short breaks (1–3 min) were provided in which participants remained seated but took a moment to shift position. One longer break (10–15 min) was provided at roughly the mid-point, in which participants were disconnected from the recording system and given time to walk around and have a snack break.

### Electrode placement and electroencephalogram recording

Electroencephalogram recordings were obtained using a 32-channel electrode cap that incorporates a subset of the International 10–20 system (Jasper, 1958). Additionally, electrodes were placed over the left and right mastoids (LM and RM, respectively). The tip of the nose was used as the reference electrode during recordings. F7 and F8 electrode sites were used in a bipolar configuration to monitor the horizontal electro-oculogram (HEOG). FP1 and an electrode placed below the left eye were used in a bipolar configuration to monitor the vertical electro-oculogram (VEOG). All impedances were maintained below 5 kΩ. The EEG and EOG were digitized (Neuroscan SynAmps amplifier, Compumedics Corp., El Paso, TX, USA) at a sampling rate of 500 Hz (0.05–100 Hz bandpass). EEG was then filtered offline (Butterworth, zero phase shift) with a lowpass designation of 30 Hz. Artifact rejection was set to exclude activity exceeding 100 μV after EEG epochs were baseline corrected. Epochs were 600 ms in duration, starting 100 ms pre-stimulus onset and ending 500 ms post-stimulus onset.

### Data reduction and analysis

ERPs evoked by each stimulus type was separately averaged together. Responses evoked by the A tone immediately following the B tone were excluded from analysis. Approximately 10% of the overall epochs were rejected due to artifact. The MMN component was statistically measured using a 40 ms window centered on the peak obtained at the mastoid electrodes in the grand-mean difference waveforms. The inversion at the mastoid that is typically observed for the MMN component was used to obtain a peak measurement because of overlap at frontal electrode sites (e.g., Fz) with the N2 component in the sound task conditions. Fz was used in the Attend-Video condition. The peak of 152 ms was used for the Attend-Pitch and Attend-Pattern conditions for all stimulus types. In the Attend-Video conditions, the peak was 174 ms for all stimulus types. The N2 component was measured using a 40 ms window centered on the peak in the grand-mean difference waveform peak at the Cz electrode (greatest S/N ratio), with a peak latency of 218 ms in the Attend-Pattern condition, and 202 ms in the Attend-Pitch condition. The P3b component was measured using a 50 ms window centered on the peak of the grand-mean difference waveform at the Pz electrode (greatest S/N ratio), with a peak latency of 364 ms in the Attend-Pattern condition, and 354 ms in the Attend-Pitch condition. No attention-related components were elicited in the Attend-Video condition because the sounds were ignored.

To statistically verify the presence of the MMN, N2, and P3b components, one-sample *t*-tests were conducted to determine whether the mean amplitude at the electrode of greatest signal-to-noise ratio for each component (Fz for MMN, Fz for target-N2, and Pz for P3b) was significantly greater than zero. To compare amplitudes of the components across conditions, repeated-measures analysis of variance (ANOVA) was calculated and Huynh–Feldt corrections were reported as appropriate. Fisher’s least significant difference (LSD) test was performed for all *post hoc* calculations. Hits, misses, false alarms, and correct rejections were calculated to determine accuracy of behavioral responses to the target tones. Button press responses were considered correct if they occurred between 100 and 900 ms from target stimulus onset. Student’s *t-*test for dependent measures was used to determine whether the hit rate (HR), false alarm rate (FAR), and reaction time (RT) differed between target types (Attend-Pitch vs. Attend-Pattern).

## Results

### Behavioral results

Hit rate to targets was high in both the Attend-Pattern (*M* = 0.93, SD = 0.07) and Attend-Pitch (*M* = 0.95, SD = 0.04) conditions, with no significant difference between them (*t*_9_ = 1.03, *p* = 0.33). FAR was low in both conditions (*M* = 0.001, SD < 0.01) and also did not significantly differ by task (*t*_9_ < 1, *p* = 0.80). RT to targets in the Attend-Pattern (*M* = 463, SD = 84) and Attend-Pitch (*M* = 463, SD = 86) conditions also did not significantly differ by task (*t*_9_ < 1, *p* = 0.96). Overall, there were no task effects on HR, RT, or FAR.

### ERP results

**Figure 2:**
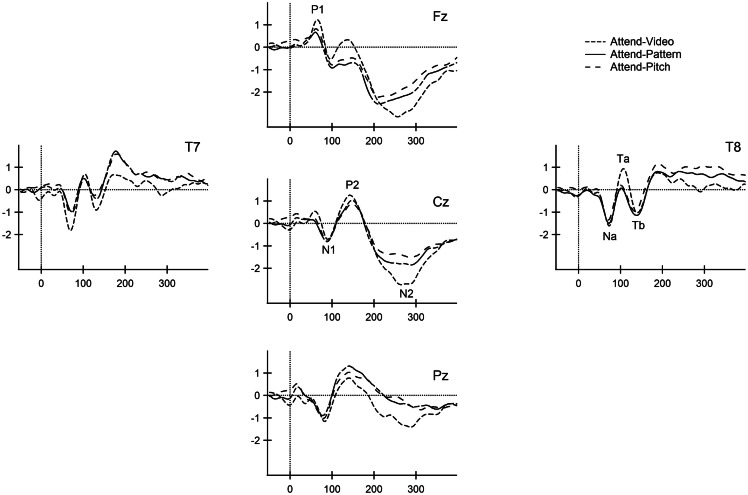
**Standard obligatory responses for all conditions**. The ERP responses to the frequently occurring tone (880 Hz) are overlain at the midline (Fz, Cz, Pz) and lateral (T7, T8) electrodes for the Attend-Video (short dashed line), Attend-Pattern (solid line), and Attend-Pitch (long dashed line) conditions. Obligatory ERP components are labeled at the electrode of greatest signal-to-noise ratio for the midline (P1, N1, P2, and N2) and the *t*-complex (Na, Ta, and Tb). x-axis is displayed in milliseconds. y-axis is the amplitude in microvolts.

Figure 2 displays the ERPs elicited by the standard stimuli, overlaying the responses from each condition of attention. In adolescence, the P1 (peak∼68 ms), N1 (peak∼90 ms), P2 (peak∼144 ms), and the obligatory-N2 (peak∼276 ms) were observed in the standard waveforms. Observed peak latencies and amplitudes of the obligatory components are consistent with their age (Ponton et al., 2000; Sussman et al., 2008).

**Figure 3:**
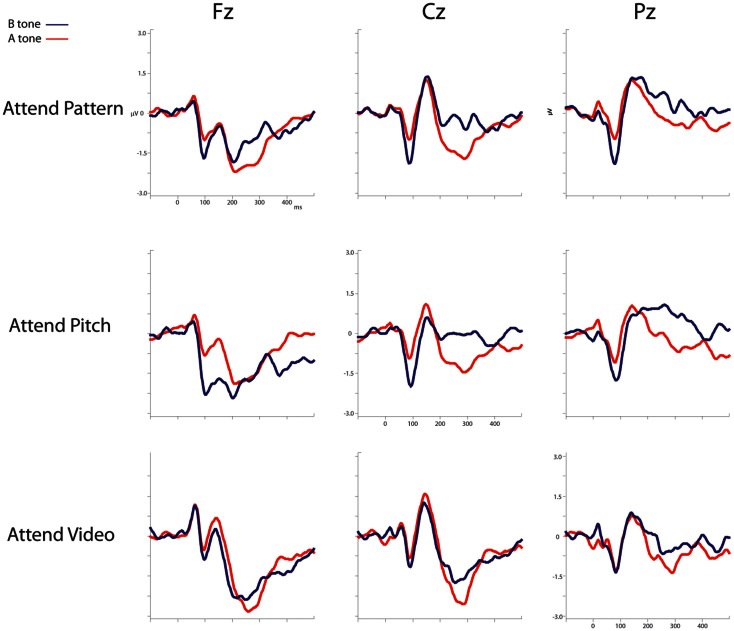
**ERPs evoked by the A and B tones**. The ERP responses evoked by the A tones (solid red line) and the B tones (solid blue line) are overlain and displayed for the Attend-Pattern (top row), Attend-Pitch (middle row), and Attend-Video (bottom row) conditions. Midline electrodes are in columns (Fz, left; Cz, middle; and Pz, right). Ordinate is the amplitude in microvolts, and the abscissa shows the timeline in milliseconds.

Figure 3 displays the ERPs evoked by the A and B tones overlain for the Attend-Pattern, Attend-Pitch, and Attend-Video conditions at the midline (Fz, Cz, and Pz) electrodes. Table 1 provides the mean amplitudes of the difference (response to B-minus-response to A) waveforms for the ERP components, and statistical presence of MMN, target-N2, and P3b components.

**Table 1:** **ERP component mean amplitudes**.

Attend/stimulus	Mean	SD	*t*-value	* ≤0.05; ** ≤0.01; ns, not sig.
**MMN (Fz)**				
Pattern/**Target**	−4.85	3.66	−4.19	**
Pattern/B	−0.23	1.45	−0.51	ns
Pitch/**Target**	−4.25	1.93	−6.96	**
Pitch/B	−1.43	1.07	−4.24	**
Video/**Target**	−3.51	4.30	−2.58	*
Video/B	−0.87	1.32	−2.08	*
**N2 (Fz)**				
Pattern/**Target**	−7.63	5.12	−4.71	**
Pattern/B	0.53	1.44	1.16	ns
Pitch/**Target**	−7.15	4.42	−5.11	**
Pitch/B	−0.47	1.31	−1.13	ns
**P3b (Pz)**				
Pattern/**Target**	16.02	10.46	4.84	**
Pattern/B	0.44	1.00	1.38	ns
Pitch/**Target**	14.98	9.90	4.79	**
Pitch/B	0.71	1.31	1.71	ns

**Figure 4:**
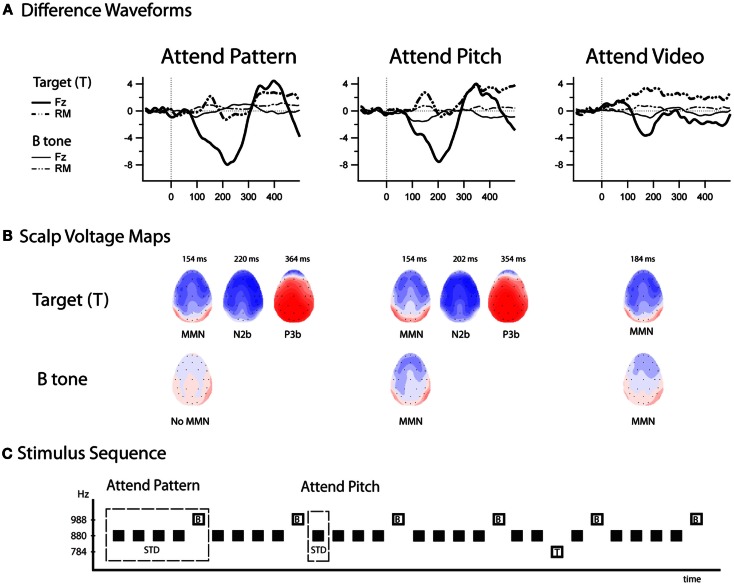
**Difference waveforms and scalp voltage maps for all conditions**. **(A)** Difference waveforms (top row) are displayed for the target tones (thick lines) and B tones (thin lines) at Fz (solid line) and the left mastoid (LM, dashed lines) in the Attend-Pattern (left column), Attend-Pitch (middle column), and Attend-Video (right column). **(B)** Scalp voltage maps (middle row) are displayed at the peak of the ERP components (peak latency above the head shots, and the ERP components labeled below). No MMN was elicited by the B tone in the Attend-Pattern condition. Target-N2 and P3b components were not elicited in the Attend-Video condition, in which the sounds were ignored. **(C)** Stimulus sequence (bottom row) is displayed as a reminder of the stimulus-eliciting tones. “T” denotes the target tones when sounds were attended and “B” denotes the fifth tone of the pattern.

Figure 4 displays the difference waveforms (ERP response to the B tone-minus-ERP response to the A tone) and voltage maps. MMNs were elicited by the target tones in all conditions (Table 1). MMNs were elicited by the B tones (the final tone of the standard repeating pattern) in the Attend-Pitch and in the Attend-Video conditions, but not in the Attend-Pattern condition (Table 1). The amplitudes of the MMNs elicited by the target tones were larger than the MMN elicited by the B tones (*F*_1,9_ = 32.13, *p* < 0.001). To determine whether there was an effect of task on MMN, a two-way repeated-measures ANOVA was conducted on the difference waveforms with factors of Attention (Pattern/Pitch/Video) and Electrode (Fz, F3, F4). There was no effect of attention on MMN amplitude for the target tones (*F*_2,18_ < 1, *p* = 0.42), nor was there a topographic difference (no main effect of electrode: *F*_2,18_ = 1, *i* = 0.38), and no interaction. In contrast, there was a main effect of attention on B tones (*F*_2,18_ = 4.96, ε = 0.86, *p* = 0.019). *Post hoc* calculation showed that MMN amplitude in the Attend-Pitch condition (−1.47 μV) was larger than that in the Attend-Video condition (−0.80 μV), and both MMNs were larger than the amplitude evoked in the Attend-Pattern condition (−0.26 μV), where there was no significant MMN.

Target-N2 and P3b components were elicited only to target stimuli (Table 1; Figure 4) when either the pattern or the pitch of the sounds was attended (Attend-Pattern and Attend-Pitch conditions), but not when the sounds were ignored (Attend-Video condition), as would be expected. For the target-N2, two-way repeated-measures ANOVA were conducted on the difference waveforms with factors of attention (pattern vs. pitch) and electrode (Fz, F3, F4, Cz, C3, C4) to assess task effects. There was no difference in amplitude according to task (no main effect of attention, *F*_1,9_ < 1, *p* = 0.92), and no main effect of electrode after correction (*F*_5,45_ = 2.83, ε = 0.52, *p* = 0.067), indicating that the target-N2 had a fronto-central distribution in adolescence. For the target-P3b, a two-way repeated-measures ANOVA was conducted on the difference waveforms with factors of attention (pattern vs. pitch) and electrode (Pz, P3, P4) to assess task effects. Similarly, task had no effect on the target-P3b (no main effect of attention, *F*_1,9_ < 1, *p* = 0.70). However, there was a main effect of electrode (*F*_2,18_ = 7.15, ε = 0.99, *p* = 0.005). *Post hoc* calculations showed that the amplitude was largest at the Pz electrode, which is a distribution that is similar to adults.

## Discussion

The goal of the study was to assess, in adolescence, task-specific modulation of neural activity resulting from selectively attending to either the pitch or the pattern of sounds in a sequence. In all conditions, the sound sequences were presented in a fixed temporal order (AAAABAAAAB …), and only the task instructions differed. The main finding was that the task performance modulated the brain’s response to the sound. This was demonstrated by the MMN response to the B tones under the different task conditions. When the pitches of the sounds were relevant to performing the task, MMN was elicited by the infrequently occurring B tones. In contrast, when the pattern of sounds was relevant to perform the task, no MMN was elicited by the B tones, as they were an integral part of the repeating standard pattern. That is, the same regularly occurring B tones evoked different brain responses depending on their relevance in performing the task. These results thus demonstrate that selective attention alters neural activity, adapting the neural response to the pertinent elements of the input required for performing a task: in this case, adapting to a five-tone standard in one condition and to single tone standard in the other.

The target 784 Hz tone was a deviant in both conditions, even though in the Attend-Pattern condition the target tone was a “pattern deviant” and in the Attend-Pitch condition it was a “pitch deviant.” MMN, target-N2, and P3b components were elicited by the targets. In the current study, in the adolescents, the target-detection N2 component had a more fronto-central scalp distribution than the more centro-parietal distribution generally found in adults. This could be due to overlap of multiple N2 components in adolescents, if, for example, anterior cingulate cortex were more involved in performing the task. This would be consistent with continued changes occurring in prefrontal cortex through adolescence (Blakemore and Choudhury, 2006). The target-P3b component, on the other hand, had a similar topography to that found in adults, with its maximal peak at the Pz electrode (Friedman and Simpson, 1994; Fabiani et al., 1998).

In contrast, the B tones had a functionally different role in the two conditions depending on whether the listener was detecting separate pitches, or the sequential five-tone pattern. The B tones were used to perform the task but were not target tones, and required no button presses in any of the three conditions. The B tones were deviants in the Attend-Pitch condition, on the basis of probability of occurrence (Sussman et al., 2003a), and MMN was elicited by them. The absence of MMN to the B tones in the Attend-Pattern condition can be explained by their function in the sequence as a part of the repeating standard pattern: they were not deviant. Thus, the way in which the sounds were used to perform the task altered the repeating standard that was maintained in memory, which modulated the change-detection process (Sussman, 2007).

When participants had no task with the sounds (Attend-Video condition), MMNs were elicited by both the B tones and the target (784 Hz) tones. This suggests that the regularity of the sequence was not automatically detected, which may be explained solely by the stimulus rate. The 575 ms onset-to-onset pace may have been too slow for automatic detection of the pattern while attending the video (Sussman and Gumenyuk, 2005; Wang et al., 2009). An alternative explanation, in conjunction with the slow presentation rate, is that MMN elicitation to B tones occurred because the patterning of the sequence was irrelevant to the task (watching a video), whether or not the B tones were detected as occurring regularly. This would suggest that MMN was elicited strictly on the basis of the ratio of tone frequencies within the sound sequence (Sussman et al., 2002; Sussman and Gumenyuk, 2005), with the A tones occurring frequently (standard) and the B tones and target tones both infrequently (deviants) (Sussman et al., 2003a).

Further, these results are consistent with previous studies using similar paradigms in adult participants (Sussman et al., 1998, 2002; Sussman and Gumenyuk, 2005), indicating that top-down processes in adolescence have similar modulatory effects in facilitating task goals. Even though the obligatory cortical ERPs in adolescents are not yet adult-like, they differ both in morphology and scalp distribution compared to adults (Sussman et al., 2008), task effects evoking the MMN component appear to be similar. This suggests that the MMN component, which is driven by the internal state of the individual, reflects aspects of auditory cognition not strictly bound to cortical maturation, which is consistent with findings of MMN elicitation in infants and toddlers (Shafer et al., 2000; Huotilainen et al., 2003).

Finally, the ability to selectively attend to sounds and ignore irrelevant sounds in the environment has important implications for clinical populations. The paradigm of the current study may be useful for testing various clinical populations. This is because changes in the neurophysiologic responses can be attributed to attentional control and not to differences in the physical characteristics of the stimuli or to the stimulus presentation rate, since the physical auditory input was the same in all conditions. This protocol may thus provide a unique way to assess attention deficits in various neurodevelopmental disorders.

### Summary

The same tone evoked different change-detection responses specific to the task, not the stimulus input. This is consistent with studies demonstrating task-specific effects in the visual system (Beck and Kastner, 2009), and consistent with idea that attention is an “emergent property” of competitive interactions with stimulus-driven processes (Desimone and Duncan, 1995). In the current study, even though the stimulus-driven input was biased toward the patterned sequence, attention mediated the neural activity to support performance goals. Task-dependent facilitation was demonstrated in that the neural response adapted either to the single tone standard or to the five-tone pattern standard inherent in the sequence. Overall, the results show, in adolescence as in adults, that attention plays an important role in modulating neural activity to facilitate performance, adjusting the time scale of adaptation dependent upon task goals.

## Conflict of Interest Statement

The authors declare that the research was conducted in the absence of any commercial or financial relationships that could be construed as a potential conflict of interest.
